# Mortality Predictors in Short-Term Mechanical Circulatory Support as a Bridge to Heart Transplantation

**DOI:** 10.3390/biomedicines13081959

**Published:** 2025-08-12

**Authors:** Carlos Domínguez-Massa, Manuel Pérez-Guillén, Iratxe Zarragoikoetxea-Jauregui, Eduardo Tébar-Botí, María José Dalmau-Sorlí, Salvador Torregrosa-Puerta, Francisco José Valera-Martínez, Claudia Marissa Aguirre-Ramón, Alexandra Margoth Merino-Orozco, Gerardo Andrés Diéguez-Palacios, Raquel López-Vilella, Ricardo Gimeno-Costa, Juan Bautista Martínez-León

**Affiliations:** 1 Department of Cardiovascular Surgery, University and Polytechnic Hospital La Fe, Avenida de Fernando Abril Martorell 106, 46026 Valencia, Spain; 2 Department of Anesthesiology, University and Polytechnic Hospital La Fe, Avenida de Fernando Abril Martorell 106, 46026 Valencia, Spain; 3 Department of Cardiology, University and Polytechnic Hospital La Fe, Avenida de Fernando Abril Martorell 106, 46026 Valencia, Spain; 4 Intensive Care Unit, University and Polytechnic Hospital La Fe, Avenida de Fernando Abril Martorell 106, 46026 Valencia, Spain; 5 Department of Surgery, University of Valencia, Avenida de Blasco Ibáñez 15, 46010 Valencia, Spain

**Keywords:** heart failure, heart transplantation, devices, extracorporeal membrane oxygenation, prognostic factors, mortality

## Abstract

**Background/Objectives**: This study evaluates the outcomes of extracorporeal membrane oxygenation (ECMO), in venoarterial configuration, and short-term ventricular assist devices (VADs) used as a bridge to heart transplantation (HT). The primary objective was to identify predictors of in-hospital mortality among patients on the urgent HT waiting list receiving short-term mechanical circulatory support, including direct ECMO-to-HT, direct short-term VAD-to-HT, and ECMO as a bridge to short-term VAD followed by HT (ECMO bridge-to-bridge). Secondary objectives included identifying predictors of in-hospital mortality in transplanted patients and assessing their long-term survival. **Methods**: A single-center, retrospective, observational, and analytical study conducted at a tertiary care hospital, including patients supported with ECMO and short-term VAD support as a bridge to HT between 2007 and 2024. **Results**: A total of 183 patients were included: 110 in the ECMO-to-HT group, 51 in the VAD-to-HT group, and 22 in the ECMO bridge-to-bridge group. Among them, 147 underwent HT (80.3%). Overall in-hospital mortality was 37.2% (115 of 183 patients survived), while in-hospital mortality among transplanted patients was 21.8% (115 of 147 survived). Independent predictors of in-hospital mortality included infection, ECMO bridge-to-bridge strategy, higher body mass index (BMI), older age, and neurological complications. In the transplanted subgroup, predictors of both in-hospital and long-term mortality were ECMO support and older recipient age. Notably, a donor BMI exceeding that of the recipient by more than 10% was associated with improved survival. **Conclusions**: The complexity of patients requiring mechanical circulatory support and the physiological effects of different devices necessitate early, individualized management based on the etiology of cardiogenic shock and urgency status.

## 1. Introduction

Despite significant advancements in ventricular assist devices (VADs), heart transplantation (HT) remains the gold standard treatment for patients with end-stage heart failure [1,2]. However, the limited availability of donor organs has lead to prolonged waiting times for HT and an increased need for mechanical circulatory support as a bridge to urgent transplantation. Among patients presenting with cardiogenic shock, extracorporeal membrane oxygenation (ECMO) is commonly utilized to provide both biventricular and respiratory support. More recently, short-term VADs have emerged as viable alternatives, either as direct bridges to HT or following initial ECMO support [3,4,5].

In Spain, a relatively high organ donation rate and unique healthcare system factors have led to the widespread use of ECMO and short-term VADs as bridges to urgent transplantation depending on the patient’s functional status and the presence of uni- or biventricular involvement. Nonetheless, these temporary support strategies carry significant risks that may increase morbidity and mortality. Despite growing clinical use, the literature on ECMO followed by short-term VAD as a bridge-to-bridge strategy to HT is limited. Given the complexity of clinical decision-making in this context, we aimed to evaluate outcomes associated with the use of venoarterial ECMO and short-term VADs, specifically CentriMag (Abbott, Chicago, IL, USA) and Impella (Abiomed, Danvers, MA, USA), as bridges to HT. The primary objective was to identify predictors of in-hospital mortality among patients on the urgent HT waiting list supported with short-term mechanical circulatory devices, including direct ECMO, direct short-term VADs (such as CentriMag and Impella), and ECMO as a bridge to specified short-term VADs. Secondary objectives included identifying predictors of in-hospital mortality exclusively in transplanted patients and evaluating their long-term survival.

## 2. Methods

### 2.1. Study Design

This was a single-center, retrospective, observational, and analytical study conducted at a tertiary care hospital between July 2007 and June 2024. We included all patients listed for HT under urgent status or Code 0 designation (national priority for the first available donor heart) who received short-term mechanical circulatory support. This included venoarterial ECMO as direct support to HT (ECMO-to-HT), as well as short-term VADs, specifically CentriMag or Impella, as direct bridges to HT (VAD-to-HT) or following prior ECMO support (ECMO bridge-to-bridge).

Patients in whom a VAD was implanted for ventricular unloading during ECMO support without successful weaning from ECMO were categorized in the ECMO-to-HT group. This classification includes patients who reached HT while still on ECMO, including those with ECPELLA configurations (combined ECMO and Impella for left ventricular unloading). In contrast, patients who received a VAD after ECMO initiation and were successfully weaned from ECMO were classified in the ECMO bridge-to-bridge group. Patients cannulated at external centers and subsequently transferred, as well those undergoing re-transplantation, were also included. Exclusion criteria included patients who received other types of devices, such as long-term implantable left VADs or total artificial hearts (e.g., SynCardia; Syncardia Systems, Tucson, AZ, USA), as a bridge to HT, as well as those who received short-term VADs as a bridge to long-term VAD implantation. Patients undergoing combined organ transplantation (e.g., heart–kidney transplantation) were also excluded.

This study was approved by the institutional ethics committee.

The following variables were analyzed: age, sex, height, weight, body mass index (BMI), and predicted left ventricular mass (LVM); comorbidities including diabetes mellitus, dyslipidemia, hypertension, and chronic obstructive pulmonary disease (COPD); device strategy (VAD-to-HT, ECMO-to-HT, or ECMO bridge-to-bridge); underlying etiology or indication for device implantation; INTERMACS (Interagency Registry for Mechanically Assisted Circulatory Support) profile; peri-implant cardiac arrest; duration of support (ECMO, VAD, or both); and laboratory parameters including lactate, creatinine, and bilirubin measured at device implantation, 48 h post-implantation, and, when applicable, at the time of HT. The occurrence of complications was also recorded, including device-related bleeding, major infections (pneumonia, bacteremia, or sepsis, defined either as infection associated with organ dysfunction or as septic shock characterized by hypotension or elevated lactate levels requiring vasopressor support), neurological complications (intracranial hemorrhage, ischemic stroke, seizures, or hypoxic–ischemic brain injury, diagnosed by imaging of functional tests), duration of invasive mechanical ventilation (IMV), and need for hemodialysis.

For transplanted patients, donor characteristics, organ ischemia time, and in-hospital mortality were assessed. Predicted LVM was calculated using the formula $\alpha \times {height\;in\;meters}^{0.54}\times {weight\;in\;kilograms}^{0.61}$, where α = 8.25 for males and 6.82 for females [6]. Donor–recipient mismatch was evaluated in terms of BMI ratio (donor BMI relative to recipient BMI) and LVM ratio (donor LVM relative to recipient LVM). Long-term survival was assessed from the date of transplantation to the date of death or last follow-up.

### 2.2. Surgical Technique

For venoarterial ECMO implantation, surgical cannulation was performed in the Intensive Care Unit to avoid transferring unstable patients, tipically using a femoro-femoral configuration with counter-incision to facilitate wound management. In cases of small femoral artery diameter or when axillary artery access was preferred, a Dacron graft was interposed. Axillary cannulation also involved venous drainage via percutaneous femoral access. During cardiac arrest, a fully percutaneous femoro-femoral cannulation was performed under echographic guidance. When the patient’s hemodynamic status allowed (e.g., advanced INTERMACS 2-3), a jugulo-axillary configuration was employed, involving surgical cannulation of the axillary artery and echographically guided insertion of the venous cannula into the jugular vein, advanced toward the inferior vena cava under fluoroscopic control [7,8].

Regarding VADs, initial left-sided support with the CentriMag system (a paracorporeal centrifugal pump offering prolonged circulatory support and adaptable to multiple configurations) was achieved via median sternotomy, using an apical left ventricular inflow cannula and an ascending aorta outflow cannula. For biventricular support, an additional right atrial drainage and pulmonary artery return cannula were added. The surgical approach later evolved to a less invasive strategy, with left thoracotomy for left ventricular inflow and subclavicular access to the axillary artery for outflow. Right ventricular support was achieved percutaneously, using femoral vein access for right atrial inflow and jugular vein access for pulmonary artery outflow. More recently, combined support has involved the use of a left-sided Impella device (an axial flow transaortic pump that unloads the left ventricle and enhances systemic and myocardial perfusion) together with right-sided CentriMag support via a dual-lumen ProtekDuo cannula (LivaNova, PLC, UK) inserted through the right internal jugular vein [9].

Prior to device implantation, an initial dose of unfractionated heparin (1 mg/kg) was administered, followed by anticoagulation management guided by anti-Xa levels and aPTT. Hemolysis markers and D-dimer were monitored every five days. A rehabilitation program was essential during the wait for transplantation, with invasive mechanical ventilation withdrawn as early as possible and active patient mobilization allowed according to the implanted device.

### 2.3. Statistical Analysis

Quantitative variables were expressed as mean and standard deviation or as median and interquartile range, depending on the normality of the distribution. Categorical variables were reported as absolute frequencies and percentages. For the bivariate analysis of continuous variables, the Student’s *t*-test was used for normally distributed data, while the Mann–Whitney *U* test was applied for non-normally distributed data. Categorical variables were analyzed using the chi-square test or Fisher’s exact test, as appropriate.

Predictors of in-hospital mortality were identified using a binary logistic regression. Long-term survival was analyzed descriptively using Kaplan–Meier curves, followed by Cox proportional hazards modeling. For model development, all variables with a *p*-value < 0.2 in the bivariate analysis, or <0.3 if considered clinically relevant, were initially included in the multivariable models. Backward stepwise selection was applied to determine the final model for both the primary outcome (in-hospital mortality of the entire cohort) and long-term survival. For the multivariable logistic regression assessing the secondary endpoint (in-hospital mortality among transplanted patients), forward stepwise selection was used to minimize overfitting given the limited number of events in this subgroup.

The reason for employing the backward stepwise selection method for the primary outcome was that it is generally recommended when considering the full correlation structure among predictors, which is particularly important in the presence of multicollinearity or confounding effects. Additionally, this approach reduces the risk of omitting relevant variables. Conversely, forward selection was used for the secondary outcome due to the limited number of events. This strategy aims to avoid overfitting, which may arise with backward selection methods that begin with all variables included, especially when events are scarce. Forward selection offers the advantage of incorporating only statistically significant variables, halting the inclusion process when no additional variables meet the predefined entry criteria. However, it presents the limitation of not accounting for all potential interactions among variables.

For handling missing data, the listwise deletion strategy was employed, as Little’s test confirmed that the missing data mechanism was MCAR (missing completely at random), with a non-significant chi-squared statistic. All statistical analyses were conducted using IBM SPSS Statistics, version 27 (IBM Corp., Armonk, NY, USA). A *p*-value < 0.05 was considered statistically significant.

## 3. Results

**Figure 1:**
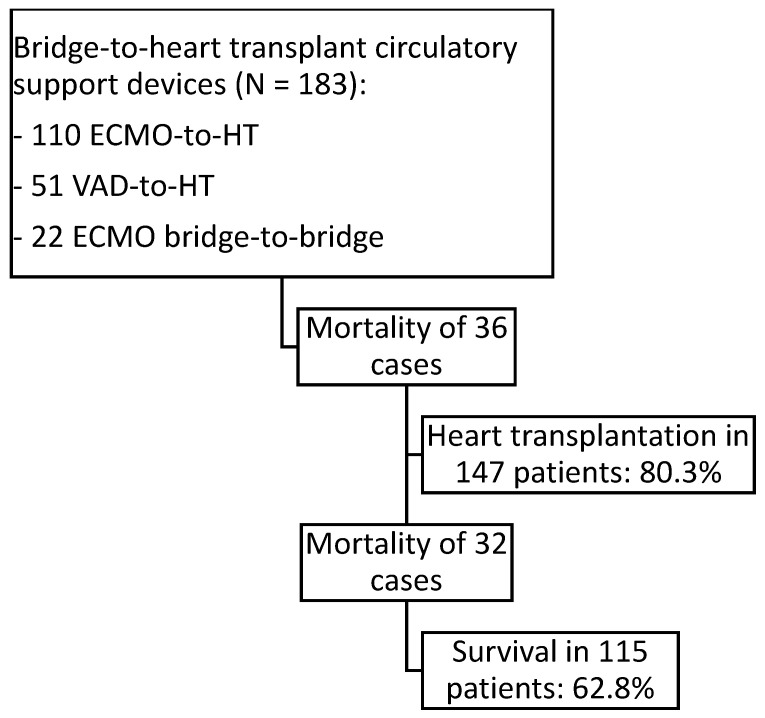
Flowchart of patients listed for urgent heart transplantation under short-term circulatory support: ECMO as direct support to HT (ECMO-to-HT) in venoarterial configuration, short-term VADs CentriMag or Impella as direct bridges to HT (VAD-to-HT), and short-term VADs CentriMag or Impella prior ECMO support (ECMO bridge-to-bridge).

Between July 2007 and June 2024, a total of 183 patients were included in this study: 110 patients supported with ECMO-to-HT, 51 with VAD-to-HT (37 CentriMag and 14 Impella), and 22 with ECMO bridge-to-bridge (18 CentriMag and 4 Impella). Within the VAD-to-HT and ECMO bridge-to-bridge groups, isolated left-sided VAD support was used in 58 patients, 14 required biventricular support, and 1 received isolated right-sided VAD support. From the entire cohort of 183 patients, 147 patients underwent HT (80.3%). The overall in-hospital mortality for the entire cohort was 37.2% (with 115 out of 183 patients surviving), whereas in-hospital mortality among transplanted patients was 21.8% (with 115 out of 147 patients surviving) (Figure 1).

Within the ECMO-to-HT group, 100 of 110 underwent transplantation (90.9%). The in-hospital mortality rate among transplanted patients in this subgroup was 25% (75 out of 100 survived), resulting in an overall in-hospital mortality of 31.8% (75 out of 110 survived). In the VAD-to-HT group, 41 out of 51 patients underwent transplantation (80.4%). In-hospital mortality among these transplanted patients was 9.8% (37 out of 41 survived), with an overall in-hospital mortality of 27.5% (37 out of 51 survived). Conversely, only 6 of 22 patients in the ECMO bridge-to-bridge group were transplanted (27.3%). The in-hospital mortality among transplanted patients in this subgroup was 50% (3 out of 6 survived), with an overall in-hospital mortality of 86.4% (3 out of 22 survived).

### 3.1. Bivariate Analysis

Table 1 presents a comparison between deceased and surviving patients in the entire study cohort. Patients who died had a significantly higher mean BMI (25.6 vs. 24.1 kg/m^2^; *p* = 0.029) and a greater prevalence of obesity (BMI > 30 kg/m^2^) (16.9% vs. 7%; *p* = 0.037). Mortality rates differed significantly according to the type of support strategy: the ECMO-to-HT and VAD-to-HT groups exhibited lower mortality compared to the ECMO bridge-to-bridge group (*p* < 0.001). Patients classified as INTERMACS class 1 had higher mortality than those in classes 2 and 3 (72.1% vs. 54.8%; *p* = 0.02). Lactate and creatinine levels measured 48 h post-implantation were significantly elevated in the in-hospital mortality (HM) group compared to the no in-hospital mortality (No-HM) group. Hemodialysis (25% vs. 8.7%; *p* = 0.003), infection (79.5% vs. 20.5%; *p* < 0.001), and neurological complications (14.7% vs. 3.5%; *p* = 0.006) were more frequent among non-survivors. Additionally, the duration of mechanical circulatory support was longer in the HM group (median 13 vs. 8 days; *p* < 0.001).

Among transplanted patients (Table 2), the ECMO-to-HT (78.1% vs. 65.2%) and ECMO bridge-to-bridge (9.4% vs. 2.6%) groups were associated with higher mortality, whereas the VAD-to-HT group had lower mortality (12.5% vs. 32.2%) (*p* = 0.032). Lactate and bilirubin levels at the time of transplantation were significantly higher in deceased patients. Infection was more prevalent in the HM group (28.1% vs. 7%; *p* = 0.003). Duration of IMV was significantly prolonged among non-survivors. Donor characteristics, including donor BMI > 10% (relative increase > 10% compared to recipient BMI) and donor–recipient predicted LVM > 10% (relative increase > 10% in donor LVM compared to recipient), did not reach statistical significance, though a donor BMI > 10% showed a trend toward better survival and was more frequent among survivors. Post-transplant ECMO requirement was also associated with increased mortality.

### 3.2. Multivariate Analysis

**Figure 2:**
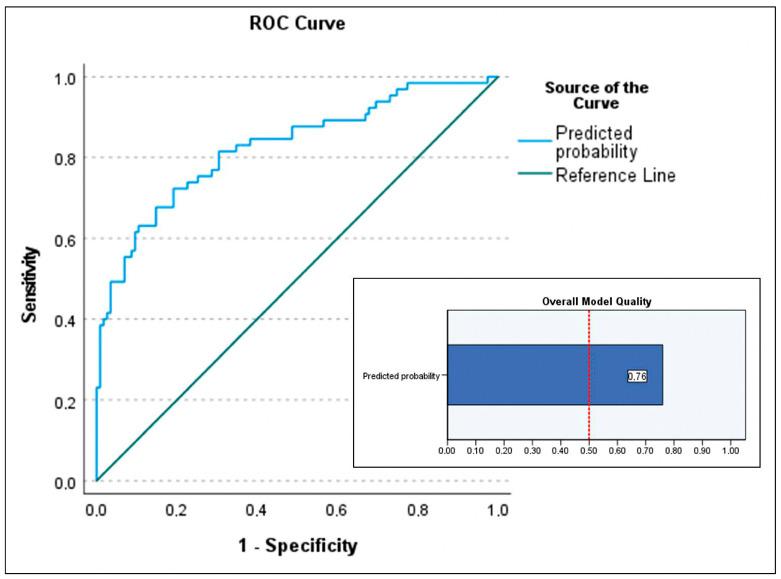
Area under the curve (AUC) of the predictive model for the primary outcome (predictive factors of in-hospital mortality in patients listed for urgent heart transplantation under short-term circulatory support) and the overall quality chart of the model. AUC of 0.826 (95% CI 0.76–0.89) and overall model quality 0.76.

**Figure 3:**
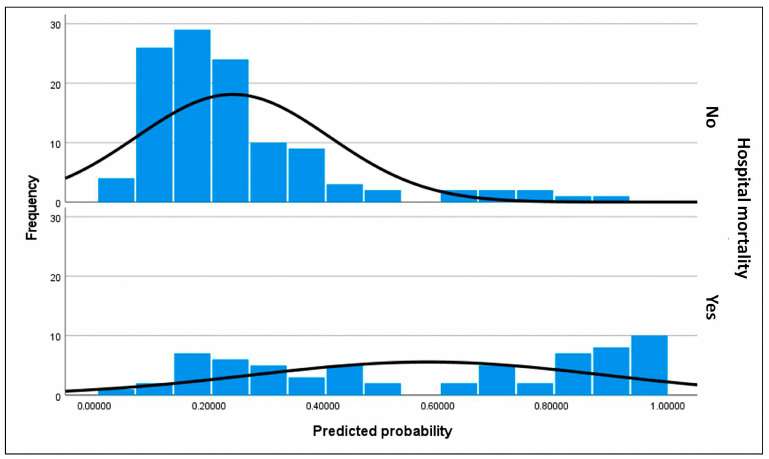
Histograms of assigned probabilities for the primary outcome (predictive factors of in-hospital mortality in patients listed for urgent heart transplantation under short-term circulatory support). The frequency corresponds to the number of subjects within each range of predicted probability. The upper histogram represents the predicted probabilities for subjects who did not experience in-hospital mortality, who constitute the majority of the cohort, whereas the lower histogram corresponds to the predicted probabilities for subjects who did experience in-hospital mortality. Predicted probabilities were calculated using the final selected model for each patient. The black curve represents a smoothed estimate of the density distribution. In the upper histogram of observed non-in-hospital mortality, nearly all surviving subjects were correctly assigned a low predicted probability, clustering at the lower end of the scale. This finding is consistent with the high specificity of the model. However, in the lower histogram of observed in-hospital mortality, a substantial proportion of subjects were also assigned a low probability, reflecting the model’s limited sensitivity. Thus, the model demonstrated greater discriminative ability for patients who survived than for those who died.

For the primary objective analysis, the initial logistic regression model included age, BMI (BMI was preferred over obesity as it is a continuous variable), device category (with VAD-to-HT as reference), implantation etiology (three indicator variables were created using chronic dilated cardiomyopathy, both ischemic and idiopathic, as the reference, compared to acute coronary syndrome, postcardiotomy shock, and other cardiopathies), INTERMACS class 1 (vs. classes 2 and 3; peri-implant cardiac arrest was excluded despite significance as it included patients in INTERMACS 1), lactate and creatinine at implantation and at 48 h, bilirubin at 48 h, hemodialysis, infection, neurological complications, and duration of support. Independent predictors of in-hospital mortality (Table 3) were infection (OR 13.979; *p* < 0.001), ECMO bridge-to-bridge (OR 12.08; *p* = 0.004), BMI (OR 1.105; *p* = 0.023), age (OR 1.041; *p* = 0.03), and neurological complications (OR 5.347; *p* = 0.039), with *p*-values determined by the Wald test. ECMO-to-HT compared to VAD-to-HT was not associated with increased mortality. If these significant predictors were used to build a predictive model for in-hospital mortality, which is beyond the scope of this study, the model would explain a high proportion of data variability with a Nagelkerke R^2^ of 0.42. The model calibration was excellent (Hosmer–Lemeshow test *p* = 0.96) but had moderate discrimination due to moderate sensitivity (53.8%) and excellent specificity (93%), resulting in a positive predictive value of 81.4%. The area under the curve for this model was 0.826 (95% CI 0.76–0.89) (Figure 2). The separation index PSEP (difference in mean predicted probabilities) was 0.341, consistent with low sensitivity and an underestimation of predicted probability of mortality among those who died (Figure 3).

**Figure 4:**
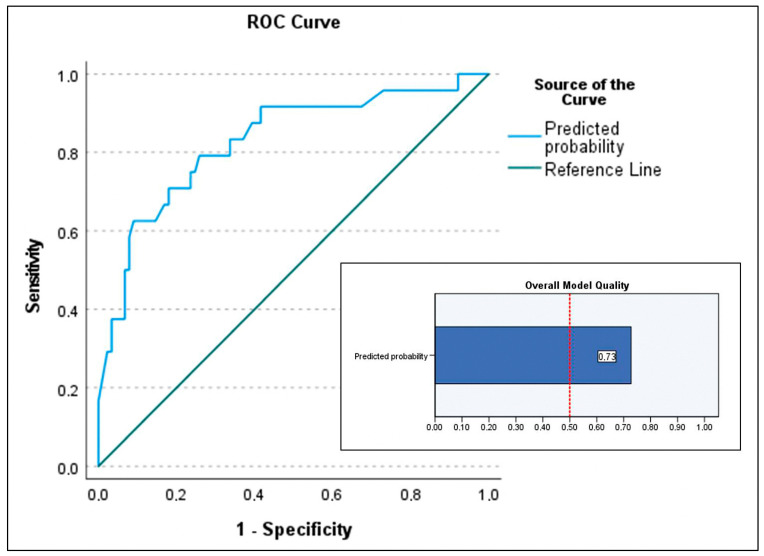
Area under the curve (AUC) of the predictive model for the secondary outcome (predictive factors of mortality in patients who underwent transplantation) and the overall quality chart of the model. AUC of 0.829 (95% CI 0.728–0.928) and overall model quality 0.73.

**Figure 5:**
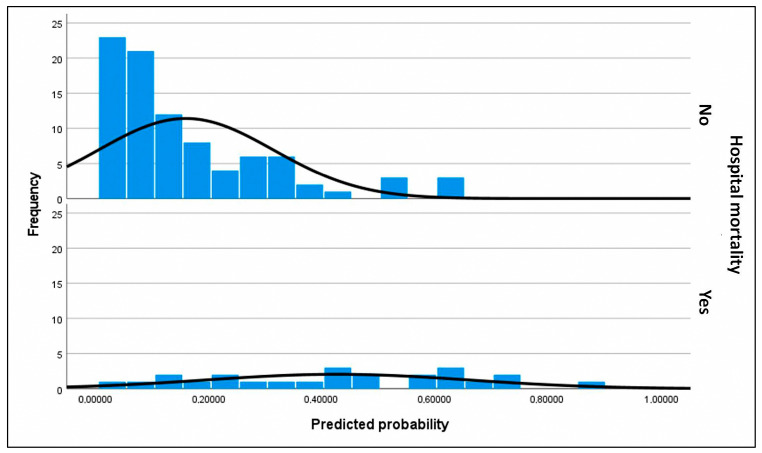
Histograms of assigned probabilities for the secondary outcome (predictive factors of mortality in patients who underwent transplantation). The frequency corresponds to the number of subjects within each range of predicted probability. The upper histogram represents the predicted probabilities for subjects who did not experience in-hospital mortality, who constitute the majority of the cohort, whereas the lower histogram corresponds to the predicted probabilities for subjects who did experience in-hospital mortality. Predicted probabilities were calculated using the final selected model for each patient. The black curve represents a smoothed estimate of the density distribution. In the upper histogram of observed non-in-hospital mortality, nearly all surviving subjects were correctly assigned a low predicted probability, clustering at the lower end of the scale. This finding is consistent with the high specificity of the model. However, in the lower histogram of observed in-hospital mortality, a substantial proportion of subjects were also assigned a low probability, reflecting the model’s limited sensitivity. Thus, the model demonstrated greater discriminative ability for patients who survived than for those who died.

For the secondary objective analyzing predictors of in-hospital mortality in patients who underwent transplantation, the maximal model included age (recipient), device category (VAD-to-HT as reference), lactate level at transplant, serum bilirubin at transplant, infection, duration of IMV, donor BMI > 10% (not significant in bivariate analysis but clinically relevant in previous studies [7]), and the need for ECMO post-transplant. Predictors of in-hospital mortality identified (Table 4) were ECMO bridge-to-bridge (OR 70.279; *p* = 0.001), ECMO-to-HT (OR 4.913; *p* = 0.015), recipient age (OR 1.13; *p* = 0.002), and donor BMI > 10% (OR 0.258; *p* = 0.015), with *p*-values from the Wald test. The Nagelkerke R^2^ was 0.344. Model calibration was acceptable (Hosmer–Lemeshow test *p* = 0.092), with low sensitivity of the selected model (37.5%) but excellent specificity (93.3%). The positive predictive value was 60%. The area under the curve was 0.829 (95% CI 0.728–0.928) (Figure 4). The PSEP was 0.268, reflecting low sensitivity (Figure 5).

### 3.3. Survival Analysis

**Figure 6:**
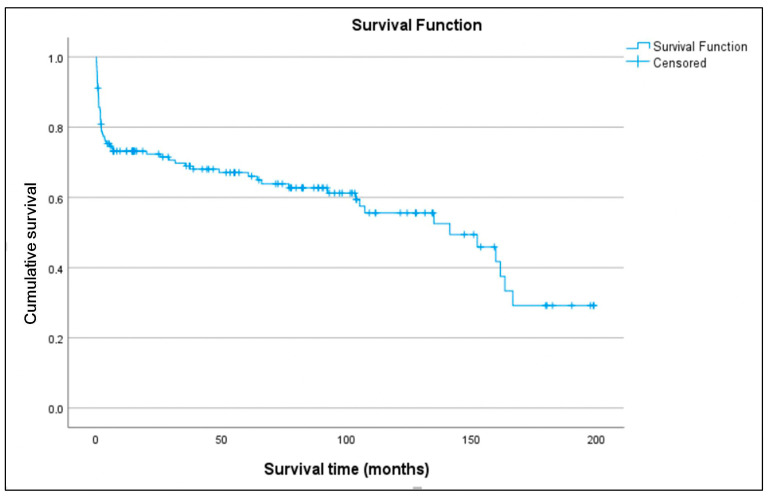
Overall survival curve of all patients who underwent transplantation.

**Figure 7:**
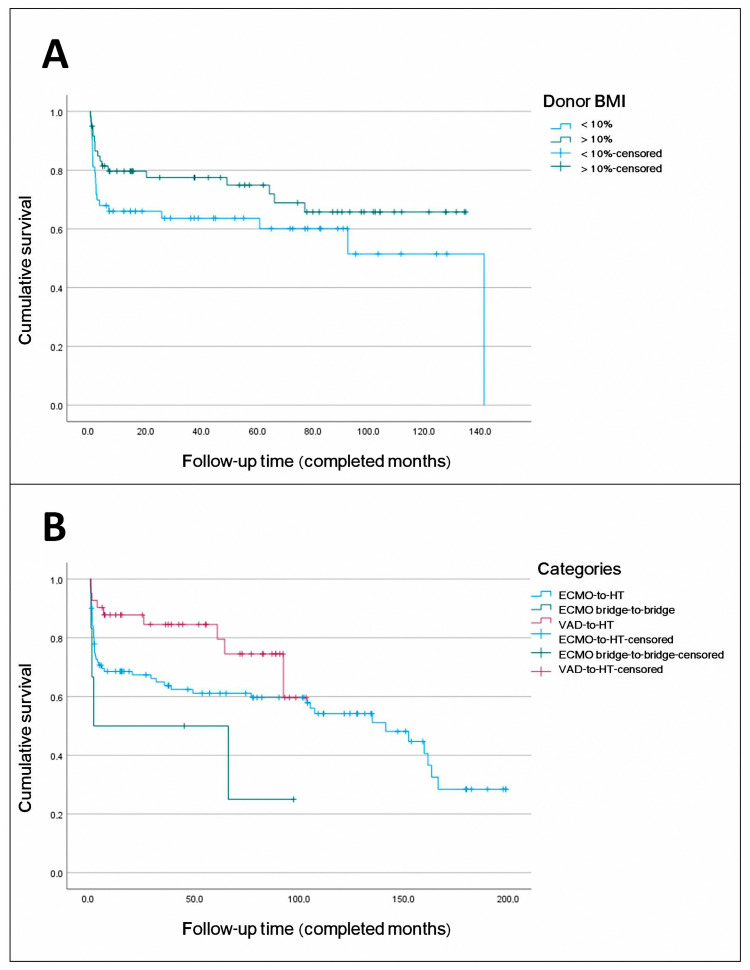
(**A**). Comparison of survival curves for donor body mass index (BMI) > 10% versus donor BMI < 10%. A total of 75% of subjects with donor BMI > 10% survived at least 49 months, while those with an increase < 10% survived at least 2 months. The estimated median survival for subjects with BMI < 10% was 141 months. Median survival for subjects with BMI > 10% could not be estimated because the survival curve did not reach 50% during follow-up. The Log-Rank test was used and was not significant (*p* = 0.15), but significance was found in the multivariate analysis. (**B**). Comparison of survival curves for device categories: ventricular assist device (VAD) as a bridge to heart transplantation (HT) versus extracorporeal membrane oxygenation (ECMO) as a bridge to HT versus ECMO bridge-to-bridge. A total of 75% of subjects with VAD-to-HT survived at least 64 months; 75% of subjects with ECMO-to-HT survived at least 2 months; and 75% of subjects with ECMO bridge-to-bridge did not survive beyond 1 month. Median survival could only be estimated for the ECMO-to-HT group (141.4 months) and the ECMO bridge-to-bridge group (1.7 months). Due to the early differences observed, the Breslow test was used and was significant (*p* = 0.028).

Long-term survival of transplanted patients was analyzed (Figure 6). Estimated survival rates at 1, 5, and 10 years were 72.8%, 66%, and 54.1%, respectively. The median survival was 141.4 months (95% CI 96.081–186.728), with the 75th percentile at 5.6 months. There were two losses to follow-up, representing 1.4% of the total cohort. The median follow-up for the entire group was 42.4 months and for the censored subgroup was 77.5 months. In univariate Cox regression analysis, variables with *p* < 0.05 included age (recipient) (*p* = 0.003), duration of support (*p* = 0.038), and need for ECMO post-transplant (*p* = 0.15). Variables with *p* < 0.3 included sex (recipient) (*p* = 0.209), BMI (recipient) (*p* = 0.106), device category (using VAD-to-HT as reference) (*p* = 0.056), and donor BMI > 10% (*p* = 0.154). These variables were included in the maximal multivariate Cox regression model except etiology or cause of implantation (*p* = 0.287), which was considered relevant for in-hospital mortality but not for long-term survival. Independent predictors of mortality identified (Table 5) (Figure 7) were ECMO bridge-to-bridge (HR 16.764; *p* < 0.001), ECMO-to-HT (HR 2.81; *p* = 0.011), recipient age (HR 1.075; *p* < 0.001), and donor BMI > 10% (HR 0.355; *p* = 0.003), with *p*-values calculated by the Wald test. The model showed a highly significant likelihood ratio test (*p* < 0.001).

## 4. Discussion

The limited availability of donor hearts has resulted in prolonged waiting times for HT, leading to an increased number of patients requiring mechanical circulatory support as a bridge to urgent HT [3,4,7,10]. In our series of 183 patients, in-hospital mortality was highest among patients supported with ECMO, with 31.8% mortality in the ECMO-to-HT group (31.8%) and a markedly elevated 86.4% in the ECMO bridge-to-bridge group, compared to 27.5% in the VAD-to-HT group. These finding suggest that patients supported by ECMO have a more severe risk profile associated with the deleterious effects of ECMO, which may increase the likelihood of clinical deterioration prior to transplantation.

Short-term VADs have emerged as effective alternatives for bridging to HT, either directly or following initial ECMO support [3,4,5]. The CentriMag, a paracorporeal centrifugal pump, offers prolonged support and can be used in multiple configurations. Bleeding, infections, and stroke remain the most common complications, with 1-year survival rates reported between 50% and 100% as a bridge to HT [11,12,13,14,15]. The Impella device is an axial flow transaortic pump that unloads the left ventricle, improving systemic and myocardial perfusion. Its less invasive surgical implantation facilitates earlier weaning from IMV and promotes active rehabilitation, with a lower complication rate compared to CentriMag. One-year survival as a bridge to HT with Impella has improved to 80–100% in various series. Different Impella devices for left ventricular support provide varying flows and durations of support: Impella CP (up to 3.7 L/min, approved for 7–10 days), Impella 5.0 (discontinued in Spain), and Impella 5.5 (up to 5.8 L/min, approved for up to 30 days). In our cohort, 6, 2, and 10 devices of each type were used, respectively. While Impella RP is available for right ventricular support, it is not employed at our center [5,16,17,18,19]. Sugimura et al. [20] analyzed 90 patients treated with Impella for cardiogenic shock predominantly due to acute myocardial infarction (61.1%). The cohort was mostly INTERMACS profile 1 (38.9%), with 73.3% requiring ECMO. In-hospital mortality was 56.7%, mainly from multiorgan failure. Ten patients underwent HT, five directly and five after long-term VAD bridging. Post-transplant in-hospital mortality was 30%. Age and lactate levels were identified as predictors of mortality.

In Spain, HT waiting list prioritization differentiates Urgency 0A (patients with full biventricular support, including ECMO) from Urgency 0B (patients with short-term univentricular support, considered lower priority). ECMO, short-term biventricular VAD, and long-term VAD with mechanical dysfunction or thromboembolic complication are considered a national maximum priority or Urgency 0A. Short-term univentricular VAD and refractory arrhythmic storm are second in the priority order or Urgency 0B [21]. Despite the relative hemodynamic stability observed in patients with an INTERMACS 3 profile, the use of a VAD is justified in those presenting risk factors for clinical deterioration, as well in INTERMACS 2 patients. This approach is supported by the beneficial effects of mechanical circulatory support in counteracting the deleterious consequences of advanced heart failure during elective HT waiting periods. In this context, jugulo-axillary ECMO implantation has also been employed, as well as the implantation of either a left VAD such as the Impella or a right VAD such as the CentriMag with the ProtekDuo dual-lumen cannula or both. The selection of each device is based on the patient’s specific clinical and anatomical characteristics as well as the presence of uni- or biventricular dysfunction. This strategy facilitates early weaning from IMV, thereby enabling active rehabilitation while awaiting HT [7,9,22,23].

The literature on short-term VAD use following ECMO bridge-to-bridge to HT remains limited [24]. ECMO offers ease of implantation and versatility for immediate biventricular and respiratory support in cardiogenic shock [25,26]. However, multiorgan failure, often due to advanced disease stage at implantation, is the leading cause of death [27,28,29,30]. ECMO increases afterload and consequently left ventricular distension, causing pulmonary edema, arrhythmias, and thrombus formation [31]. Post-HT outcomes are poorer in ECMO-supported patients compared to those supported with long-term VADs or without support [32]. Different series [3,7,33,34,35,36,37,38] report 30-day mortality ranges from 20 to 35%, with 1-year survival between 55% and 70%, generally maintained at 3 and 5 years. Disease severity (reflected in multiorgan failure scores, especially related to renal and hepatic function), lactate levels, the need for IMV at transplantation, inotropic support, advanced age, and sometimes female sex are linked to worse outcomes. In our cohort, predictors of in-hospital mortality included infection, ECMO bridge-to-bridge, higher BMI, older age, and neurological complications. Among transplanted patients, ECMO bridge-to-bridge, ECMO-to-HT, and older recipient age predicted in-hospital mortality, while donor BMI > 10% predicted survival. In transplanted patients, neurological complications were not identified as a predictor of mortality. These complications are strongly associated with the use of mechanical circulatory support devices due to embolic events, the need for anticoagulation, and the critical condition of patients, including cases of cardiac arrest at the time of device implantation. Neurological complications, although frequent in the entire cohort (14.7% of deceased), were not predictive of mortality among transplanted patients, likely because many with such complications did not survive to transplantation. Only 3.4% of those who reached HT presented with neurological complications.

Yin et al. [39] included 6206 patients with long-term VADs, 75 with short-term percutaneous VADs such as Impella and TandemHeart (Livanova, London, UK), 134 with ECMO, 38 with short-term left VADs like CentriMag, and 75 with short-term biventricular VADs like CentriMag, all undergoing HT. One-year survival was lower in the ECMO group (71.2%), followed by percutaneous VADs (79.9%), short-term biventricular VADs (86.2%), and short-term left VADs (89.5%) and was highest in long-term left VADs (89.6%). Predictors of mortality included donor age, donor–recipient sex mismatch, recipient age, recipient BMI, recipient creatinine levels, ischemic time, and use of ECMO or percutaneous VADs versus long-term left VADs. Data on pre-transplant support duration were not available for analysis. Patients on ECMO with refractory cardiogenic shock had increased multiorgan failure and perioperative complications. Despite rapid stabilization offered by ECMO, it did not improve outcomes compared to short-term biventricular VADs like CentriMag, which require more invasive surgical implantation. TandemHeart is not utilized at our institution due to its invasiveness and associated vascular complication in comparison to Impella. This paracorporeal centrifugal pump is inserted into the left atrium via a transseptal puncture for drainage purposes. It necessitates a more intricate learning curve [40,41]. In our long-term series, the predictors of mortality mirrored those identified for in-hospital mortality among transplanted patients, that is, ECMO bridge-to-bridge, ECMO-to-HT, and older recipient age, with donor BMI > 10% as a predictor of survival. Estimated survival rates at 1, 5, and 10 years were 72.8%, 66%, and 54.1%, respectively. Although not the primary focus of this study, overall HT survival at our center in recent years has been approximately 80% at 1 year, reaching up to 90% in elective transplants performed without the use of mechanical circulatory support devices.

In acute coronary syndrome, although ECMO benefits are unclear, the Danger Shock Trial showed improved mortality with early Impella support. Factors influencing outcomes include underlying pathology, uni- or biventricular dysfunction, device choice, and timing of implantation. Patients without compensatory mechanisms for chronic heart failure benefit from early circulatory support and unloading to improve myocardial perfusion, reduce filling pressures, and consequently decrease the risk of pulmonary edema. Advanced age substantially increases mortality [30,42,43,44]. The literature emphasizes early correction of organ hypoperfusion and multiorgan dysfunction, as hemodynamic restoration does not always correct the loss of hemodynamic coherence between micro- and macrocirculation, with invasive monitoring essential to reduce mortality [45,46]. Early and individualized support based on pathology and vigilant complication avoidance are crucial to improve outcomes [47].

Obesity was present in 8.8% of transplanted patients and 15.6% of deceased patients of our series, possibly reflecting selection bias, with obese patients dying before transplantation. This may explain why obesity was not a significant mortality predictor post-HT given its lower prevalence. However, in our analysis of patients who underwent HT, donor BMI > 10% emerged as a predictive factor of both short- and long-term survival. The International Society for Heart and Lung Transplantation (ISHLT) recommends donor weight within 30% of recipient weight (20% if the donor is female and recipient male) [48]. Graft undersizing increases mortality [49]. Holzhauser et al. [50] analyzed 288 transplanted patients, including 58 with long-term left VADs, reporting 90% 1-year survival. No differences were found based on donor–recipient size mismatch, even within the long-term left VAD group.

Managing mechanical support, especially ECMO, in obese patients is challenging due to anatomical and physiological factors. However, some studies report no association between obesity and ECMO mortality. Indeed, the obesity paradox has been described, where adipose tissue may confer protective cardiovascular and immune effects and serve as a metabolic reserve [51,52,53]. The impact of donor obesity is less clear but may introduce risk influencing HT outcomes. Several studies have found survival benefits associated with donor BMI exceeding recipient BMI [7,54,55]. Our study highlights the importance of obesity as a predictor of mortality in the overall cohort, while a donor BMI > 10% emerged as a protective factor. In large registries, recipient BMI has increased significantly over time; therefore, despite the discordant findings regarding its implications across different studies, it remains an important factor to consider [56].

Limitations of our study include a limited sample size and number of events, which may reduce the statistical power to estimate parameters accurately. Being a single-center study provides homogeneity but limits external validity. Likewise, due to overfitting and the slight imbalance among the categories of the variables, excellent discrimination may be observed within the cohort, but this could reduce generalizability of the results. The use of listwise deletion for handling missing data resulted in a loss of statistical power. A certain degree of selection bias may also be present, as previously noted. This is exemplified by the subgroup of obese patients who succumbed prior to undergoing HT. A similar situation applies to neurological complications, which are strongly associated with this patient population and the use of mechanical circulatory support devices. Variability in the types of devices used and HT indications could have influenced results. Patients in the ECMO bridge-to-bridge group exhibited markedly worse outcomes. This may also be attributable to selection bias. This group could represent a more critically ill population requiring prolonged circulatory support to reach HT, having corrected multiorgan failure where applicable. Moreover, when comparing the use of CentriMag versus Impella as left ventricular support exclusively in patients with VADs, a significantly higher mortality rate was observed with CentriMag (87.9% vs. 64.1%; *p* = 0.02). This finding suggests the need for an independent study of risk factors in the isolated VAD patient groups.

Additionally, the specific characteristics of the Spanish transplant program should be considered. The high donation rate in Spain significantly reduces waiting times for transplantation [57]. ECMO is commonly used as a direct bridge to HT in Spain due to the peculiarities of the donation system. In 2017, HT prioritization criteria were modified to limit ECMO use to 7–10 days depending on IMV needs, mandating transition to other strategies, but no mortality improvement was observed. In 2023, this time limitation was removed, with strict criteria applied to avoid ECMO-related complications [21].

It is noteworthy that the multivariate model for the primary outcome showed low sensitivity, with a high proportion of false negatives, i.e., it failed to identify patients who eventually died. However, it exhibited high specificity, significantly detecting those who survived, indicating a low false-positive rate. Consequently, the model had a high positive predictive value. In our context, false positives may have a higher cost than false negatives, as incorrectly predicting death for a patient who survives could lead to withdrawal of treatment and loss of a potentially salvageable patient. The high specificity and positive predictive value of our model support this approach. One of the main consequences of low sensitivity is a reduced negative predictive value. Enhancing sensitivity would ideally require a larger sample size with an increased number of outcomes events, thereby improving the statistical power to detect true-positive cases.

Finally, it should be mentioned that ECMO-to-HT compared to VAD-to-HT did not increase mortality in the overall sample analysis (primary outcome). However, after excluding five outlier subjects identified by Cook’s distance and DF betas metrics, ECMO-to-HT became significant in the multivariate model compared to VAD-to-HT (OR 3.556; *p* = 0.025). This suggests that the effect may exist but is unstable and depends on few observations. Moreover, among transplanted patients, the initial circulatory support device may lose importance since these patients arrive at transplantation in relatively good condition. Accordingly, the need for ECMO after HT was significant in bivariate analysis but not in the multivariate model.

## 5. Conclusions

In conclusion, short-term VADs have been established as viable alternatives for bridging to HT, either as direct support or following initial ECMO assistance (bridge-to-bridge strategy). Independent predictors of in-hospital mortality included infection, ECMO bridge-to-bridge strategy, elevated BMI, advanced age, and neurological complications. Among transplanted patients, both in-hospital and long-term mortality were associated with ECMO bridge-to-bridge, ECMO-to-HT, and older recipient age, whereas a donor BMI > 10% was associated with improved survival. The high mortality observed in patients undergoing ECMO bridge-to-bridge highlights the need for timely and appropriate management to prevent complications and improve outcomes. The complexity of patients requiring mechanical circulatory support, along with the distinct physiological impact of each device, demands early, individualized intervention tailored to the etiology of cardiogenic shock and the degree of urgency.

## Figures and Tables

**Table 1:** Bivariate analysis of predictors of in-hospital mortality in the total sample.

	Total (N = 183)	In-Hospital Mortality (HM) (N = 68)	No In-Hospital Mortality (No-HM) (N = 115)	*p*-Value
Demographic data				
Age (years); median (IQR)	55.5 (14)	57.6 (11.5)	53.5 (14)	0.06
Male sex	76.5%	75%	77.4%	0.712
BMI (kg/m^2^); mean (SD)	24.6 (4.5)	25.6 (4.9)	24.1 (4.1)	0.029
Obesity (BMI > 30 kg/m^2^)	10.4%	16.9%	7%	0.037
Comorbidities				
Diabetes mellitus	22.4%	22.1%	22.6%	0.931
Dyslipidemia	32.8%	35.3%	31.3%	0.578
Hypertension	41.5%	45.6%	39.1%	0.392
COPD	10.9%	10.3%	11.3%	0.832
Device and implantation data				
VAD-to-HT	27.9%	20.6%	30.2%	<0.001
ECMO-to-HT	60.1%	51.5%	65.2%	
ECMO bridge-to-bridge	12%	27.9%	2.6%	
Acute coronary syndrome	24.6%	33.8%	19.1%	0.05
Chronic dilated cardiomyopathy	62.3%	51.5%	68.7%	
Postcardiotomy shock	7.1%	10.3%	5.2%	
Other etiologies	6%	4.4%	7%	
In-hospital implantation	93.4%	92.6%	93.9%	0.738
Peri-implant cardiac arrest	16.9%	22.1%	13.9%	0.156
INTERMACS 1	61.2%	72.1%	54.8%	0.02
Time period 2007–2011	19.1%	13.2%	22.6%	0.308
Time period 2012–2016	27.9%	29.4%	27%	
Time period 2017–2021	33.3%	39.7%	29.6%	
Time period 2022–2024	19.7%	17.6%	20.9%	
Total support time (days); median (IQR)	10 (10)	13 (15)	8 (8)	<0.001
Laboratory data				
Creatinine at 48 h (mg/dL); median (IQR)	0.9 (0.6)	1.15 (1)	0.86 (0.6)	0.003
Pre-implant bilirubin (mg/dL); median (IQR)	1.4 (1.6)	1.45 (1.79)	1.4 (1.52)	0.617
Bilirubin at 48 h (mg/dL); median (IQR)	1.42 (1.59)	1.7 (2.26)	1.4 (1.39)	0.28
Complications				
Hemodialysis	14.8%	25%	8.7%	0.003
Infection	21.3%	79.5%	20.5%	<0.001
Local bleeding	15.3%	46.4%	53.6%	0.27
Neurological complications	7.7%	14.7%	3.5%	0.006

HM: In-hospital mortality. No-HM: No in-hospital mortality. IQR: Interquartile range. BMI: Body mass index. SD: Standard deviation. COPD: Chronic obstructive pulmonary disease. VAD: Ventricular assist device. ECMO: Extracorporeal membrane oxygenation. HT: Heart transplantation. INTERMACS: Interagency Registry for Mechanically Assisted Circulatory Support.

**Table 2:** Bivariate analysis of predictors of in-hospital mortality in patients who underwent heart transplantation.

	Total (N = 147)	In-Hospital Mortality (HM) (N = 32)	No In-Hospital Mortality (No-HM) (N = 115)	*p*-Value
Demographic data				
Age (years); median (IQR)	55.5 (13.2)	59 (7)	53.5 (14)	0.01
Male sex	76.2%	71.9%	77.4%	0.517
BMI (kg/m^2^); mean (SD)	24.3 (4.2)	25.2 (4.5)	24.1 (4.1)	0.155
Obesity (BMI > 30 kg/m^2^)	8.8%	15.6%	7%	0.157
Comorbidities				
Diabetes mellitus	23.1%	25%	22.6%	0.777
Dyslipidemia	33.3%	40.6%	39.1%	0.878
Hypertension	39.5%	40.6%	62.5%	0.417
COPD	10.9%	9.4%	11.3%	1
Device and implantation data				
VAD-to-HT	27.9%	12.5%	32.2%	0.032
ECMO-to-HT	68.8%	78.1%	65.2%	
ECMO bridge-to-bridge	4.1%	9.4%	2.6%	
Acute coronary syndrome	23.8%	40.6%	19.1%	0.067
Chronic dilated cardiomyopathy	63.9%	46.9%	68.7%	
Postcardiotomy shock	4.8%	3.1%	5.2%	
Other etiologies	7.5%	9.4%	7%	
Peri-implant cardiac arrest	15%	18.8%	13.9%	0.576
INTERMACS 1	57.8%	68.8%	54.8%	0.157
Time period 2007–2011	23.1%	25%	22.6%	0.986
Time period 2012–2016	27.2%	28.1%	27%	
Time period 2017–2021	29.3%	28.1%	29.6%	
Time period 2022–2024	20.4%	18.8%	20.9%	
Total support time (days); median (IQR)	8 (8)	9 (13)	8 (8)	0.555
Time in Code 0 (days); median (IQR)	5 (6)	4.5 (7)	5 (6)	0.661
Laboratory data				
Lactate at transplant (mmol/L); median (IQR)	1 (0.7)	1.35 (0.8)	0.95 (0.5)	<0.001
Creatinine at transplant (mg/dL); median (IQR)	0.78 (0.5)	0.95 (0.7)	0.73 (0.5)	0.122
Bilirubin at transplant (mg/dL); median (IQR)	1.3 (1.7)	1.45 (2.83)	1.24 (1.34)	0.021
Complications				
Hemodialysis	10.2%	15.6%	8.7%	0.319
Infection	11.6%	28.1%	7%	0.003
Neurological complications	3.4%	3.1%	3.5%	1
Duration of IMV (days); median (IQR)	3 (7)	6 (8)	2 (6)	0.002
Absence of IMV at transplant	59.6%	43.8%	64%	0.039
Transplant data				
Donor age (years); median (IQR)	47 (17.5)	47 (19.8)	48 (17.5)	0.684
Donor male sex	65.5%	50%	69.7%	0.072
Ischemia time (minutes); median (IQR)	188 (94)	183 (85)	190 (95)	0.255
Donor BMI (kg/m^2^); mean (SD)	26.4 (5.2)	26.6 (5.3)	26.2 (5.5)	0.606
Donor BMI > 10%	53.1%	37.5%	57.3%	0.084
Donor LVM > 10%	47.2%	40%	48.9%	0.474
Need for ECMO post-transplant	44.9%	65.6%	39.1%	0.008

HM: In-hospital mortality. No-HM: No in-hospital mortality. IQR: Interquartile range. SD: Standard deviation. BMI: Body mass index. COPD: Chronic obstructive pulmonary disease. VAD: Ventricular assist device. ECMO: Extracorporeal membrane oxygenation. HT: Heart transplantation. INTERMACS: Interagency Registry for Mechanically Assisted Circulatory Support. IMV: Invasive mechanical ventilation. LVM: Left ventricular mass.

**Table 3:** Multivariate analysis of the primary endpoint: predictors of in-hospital mortality in patients listed for urgent heart transplantation under short-term circulatory support.

	OR	95% CI OR	*p*-Value
Infection	13.979	4.941–39.555	<0.001
ECMO bridge-to-bridge	12.08	2.17–67.265	0.004
BMI	1.105	1.014–1.204	0.023
Age	1.041	1.004–1.079	0.03
Neurological complications	5.347	1.089–26.256	0.039

OR: Odds ratio. 95% CI: 95% confidence interval. ECMO: Extracorporeal membrane oxygenation. BMI: Body mass index.

**Table 4:** Multivariate analysis of the secondary endpoint: predictors of mortality in patients who underwent heart transplantation.

	OR	95% CI OR	*p*-Value
ECMO bridge-to-bridge	70.279	5.415–912.137	0.001
ECMO-to-HT	4.913	1.363–17.713	0.015
Recipient age	1.13	1.047–1.219	0.002
Donor BMI > 10%	0.258	0.087–0.766	0.015

OR: Odds ratio. 95% CI: 95% confidence interval. ECMO: Extracorporeal membrane oxygenation. HT: Heart transplantation. BMI: Body mass index.

**Table 5:** Multivariate Cox analysis of patients who underwent heart transplantation.

	HR	95% CI HR	*p*-Value
ECMO bridge-to-bridge	16.764	4.408–63.752	<0.001
ECMO-to-HT	2.81	1.265–6.239	0.011
Recipient age	1.075	1.036–1.116	<0.001
Donor BMI > 10%	0.355	0.178–0.707	0.003

HR: Hazard ratio. 95% CI: 95% confidence interval. ECMO: Extracorporeal membrane oxygenation. HT: Heart transplantation. BMI: Body mass index.

## Data Availability

The data underlying this article are available in the article.
